# Cognition and Academic Performance: Mediating Role of Personality Characteristics and Psychology Health

**DOI:** 10.3389/fpsyg.2021.774548

**Published:** 2021-12-07

**Authors:** Yueqi Shi, Shaowei Qu

**Affiliations:** School of Humanities and Social Sciences, University of Science and Technology Beijing, Beijing, China

**Keywords:** cognitive ability, personality characteristics, psychology health, academic performance, mediation

## Abstract

This study uses personality and psychology health characteristics of high school students as intermediary variables to study how cognitive ability affects academic performance, and analyzes memory, information processing, presentation, logical reasoning, and thinking transformation ability in high school students. In this study, the structural equation model (SEM) was used to analyze the mediating effect, and the bootstrap method was used to test the significance of the mediating effect. The participants were 572 high school students from Beijing, China. They completed a survey that included questions on cognitive ability, personality characteristics, and psychology health. This study uses structural equation modeling for mediation analysis. Through the analysis of four models of comprehensive academic performance, Chinese academic performance, mathematics academic performance, and English academic performance, the results of the study showed that cognitive ability has a significant effect on academic performance, and personality characteristics and psychology health play a partially mediating role between cognitive ability and English academic performance. The mediation effect is about 40%.

## Introduction

Research has emphasized the important role of cognitive ability in the learning process. In educational practice, subsequently, attention has been paid to the cultivation of strong cognitive abilities in students. However, a series of studies have shown that cognitive ability is not the only factor that determines the level of academic performance in students (Shao, 1983). An individual’s academic performance might not only be determined by their cognitive abilities, but also by their overall positive mental state. However, there are only a few previous studies on the mechanism of cognitive ability affecting academic performance. This study uses the personality characteristics and psychology health of high school students as mediating variables to study the influence mechanism of cognitive ability on academic performance. This study aims to identify the mediating effect of personality and psychology health characteristics on cognitive ability and academic performance, to further clarify the influencing mechanisms of cognitive ability on the academic performance of high school students.

### The Impact of Cognitive Ability on Academic Performance

Cognitive ability refers to the ability of the human brain to process, store and extract information, including processes such as attention, memory, and reasoning ability. It is the key psychological element for people to successfully complete an activity (Sternberg and Sternberg, 2009) and is currently one of the most studied and most stable predictors of academic performance (Matthias et al., 2016; Vilia et al., 2017). Previous studies have focused on the direct impact of individual-level cognitive ability on academic performance (Kuncel et al., 2004; Miriam et al., 2011). A study by Xu and Li (2015) on 4,743 junior high school students found that selective attention, short-term memory, and reasoning ability are significant predictors of linguistic and mathematics performance. Rohde and Thompson (2007) found that cognitive ability directly predicts academic performance, and the correlation between the two is as high as 0.38. Ian (2006) conducted a 5-year follow-up study of more than 70,000 British students and found that the correlation between general cognitive ability at 11 years old and academic performance at 16 years old was 0.81. Paulo used multiple regression stepwise analysis and standardized regression coefficients (*β*) to evaluate the relationship between the inference dimensions and physical and chemical achievements in each semester of the three semesters and found that reasoning ability was significantly positively correlated with students’ physical and chemical performance (Grass et al., 2017). Liu measured 499 Chinese children’s cognitive abilities such as visual space, arithmetic, and reading, and collected their mathematics and Chinese learning scores for two consecutive academic years in the same year of the cognitive test and the year after the cognitive test. Correlation analysis shows that visual space, arithmetic, and reading ability are significantly related to academic performance (Liu et al., 2021).

Although the relationship between general cognitive ability and academic performance seems clear, it is difficult to fully understand the mechanism of their complex relationship. In a learning context, the importance of cognitive abilities in human learning activities can only be more deeply reflected by including specific cognitive abilities in the scope of investigation (David, 2005) because learning activities not only involve different specific abilities, but these abilities also work together in unpredictable ways. As such, there is still no consensus on how model cognitive ability influences academic performance (Formazin et al., 2011). Zhang (2008) found that the correlation coefficients between logical reasoning ability (LRA) and Chinese and mathematics scores are all around 0.3, while the level of attention is not significantly correlated with the scores of the two subjects. However, Xu and Li (2015) found that selective attention has a significant correlation with the performance of the two subjects, and the correlation coefficient between reasoning ability and the performance of Chinese and mathematics is between 0.4 and 0.5. These results indirectly confirmed that cognitive ability only influences academic performance as a whole, and that conclusions cannot be drawn yet regarding the complex interaction of individual factors on academic performance.

### The Mediating Role of Personality Characteristics

Many studies have shown a certain correlation between students’ academic performance and their personality factors. Gerhard (1996) has confirmed through experimental studies that personality factors have a certain impact on academic performance while Leino (1989) and others believed that non-intellectual factors, including personality characteristics, were the main cause of academic performance. American scholar Gough (1964) used the “California Psychological Inventory” (CPI) to investigate 18 personality factors of middle school students and make a correlation analysis with their academic performances, and found that there were at least eight personality factors (such as the desire to dominate, sense of responsibility, socialization, tolerance, independence, etc.) that correlated significantly with academic performance. Richardson et al. (2009) measured five major personality characteristics as well as achievement motivation. They found that both, rigor and achievement motivation, could explain the changes in grade point average (GPA) for students. The impact is regulated by their achievement motivation. Adrian and Tomas (2005) also examined the internal relationship between individual personality characteristics and knowledge level and found that rigor and openness had a significant positive correlation with knowledge level. Through research, Ruffing found that general cognitive ability is positively correlated with academic performance, and there are obvious personality differences. Differences in personality characteristics can explain the incremental variance that exceeds general cognitive ability (Ruffing et al., 2015).

Although there has been prior research on the relationship between cognitive ability and personality characteristics, they were mainly based on qualitative research or correlation analyses, which led to only a few effective identifications of a causal relationship between them. Cognitive ability and personality characteristics, as the two major components of individual psychology, are both independent and related (Li and Zhang, 2015), but there are different opinions on their relevance given that cognitive ability and personality characteristics are indicators to measure different dimensions of ability (James et al., 2006). Most factors of personality characteristics and cognitive ability are very weakly correlated, so the two can be independently used as explanatory variables of individual behavior (Ackerman and Heggestad, 1997); even though some scholars believe that cognitive ability and personality characteristics will affect each other (Borghans et al., 2008). Tania’s research on personality traits has a significant impact on information processing ability (IPA) and found that different personality traits, especially conscientiousness, will reduce the impact on performance according to the increase in education level, indicating that personal personality has a greater impact on the promotion of academic achievement (Cerni et al., 2021). In recent years, the causal relationship between the two has been paid more attention. There is literature reporting the use of long-term tracking survey data to verify the causal relationship between personality characteristics and cognitive ability from theoretical and empirical aspects and found that cognitive ability can significantly affect the development of personality characteristics (Heckman et al., 2018).

### The Mediating Role of Psychology Health

Psychology health is a person’s subjective experience. It includes not only positive emotions, but also all aspects of personal life. It refers to the ability to show a positive and healthy mental state in all aspects of learning, life, interpersonal communication, and self-awareness (Hu and Xiao, 2021).

There is a general correlation between psychology health and personality characteristics, but the correlation between psychology health and personality trait factors is not completely consistent and the coefficients are different (Wang, 2021). In psychology health, personality characteristics play an important role. The theory of personality characteristics believes that personality characteristics can determine a person’s behavior. Indeed, some studies have shown that there is a high correlation between the personality characteristics of students and their psychology health. For example, the more extroverted the students are, the less likely they are to have psychology symptoms (Chu et al., 2019). The personality characteristics that affect psychology health mainly include optimism, easygoingness, trust, and enthusiasm. For example, solitary and highly sensitive students tend to be neurotic and incompatible, which is not conducive to their own development. Many studies have shown that the level of a student’s psychology health is closely related to their level of learning efficiency (Wu, 2021).

Secondly, existing studies mostly focus on the relationship between psychology health and psychological characteristics (Wang and Zhang, 2012; Wu et al., 2018), and not on the impact of psychology health on academic performance. However, academic performance is one of the important indicators of student development and educational outcomes (Wang and Jessica, 2016), as well as an important part of the psychology health mechanism (Zhang et al., 2017). Limited empirical research suggests that the psychology health of Chinese adolescents is closely related to their academic performance (Zhang and Liu, 2001).

According to the S-C-R theoretical model established by cognitive psychological theorists, it is not the stimulus itself that affects individual behavior, but our perception of the event. In this model, S (stimulus) refers to the components that can cause stimulus in the entire external world, including external events, situations, interpersonal relationships, and their own behavior; C (consciousness) refers to consciousness and experience; R (response) refers to response (Wang, 2008), therefore, students with stronger cognitive abilities will obtain more stimulus information from the outside world, and their understanding of this information will be deeper, and their judgments and responses to the outside world will be more autonomous. This judgment and response to external stimulus information can reflect the psychology health of students. So the strength of cognitive ability can significantly affect the psychology health of students.

### Research Hypothesis

This study combines the classification of cognitive ability by Wo and Lin (2000), Xu and Li (2015) and Liang et al. (2020) and divides cognitive ability into working memory ability (MA), IPA, representational ability (RA), LRA, and thinking conversion ability (TCA). It explores the specific influence of different cognitive abilities on academic performance and puts forward the following hypotheses:

*Hypothesis 1a*: MA is positively correlated with academic performance.

*Hypothesis 1b*: IPA is positively correlated with academic performance.

*Hypothesis 1c*: RA is positively correlated with academic performance.

*Hypothesis 1d*: LRA is positively correlated with academic performance.

*Hypothesis 1e*: TCA is positively correlated with academic performance.

At the same time, existing studies have focused more on the impact of individual factors of cognitive ability and personality characteristics on academic performance; thus, the correlation between cognitive ability and personality characteristics remains unclear. For the causal relationship between cognitive ability and personality characteristics, only a few empirical studies have been conducted with personality characteristics as an intermediary for the influence of cognitive ability. Therefore, this study uses personality characteristics as an intermediary variable to analyze the influence of cognitive ability on academic performance, and analyze how cognitive ability influences academic performance. We propose the following hypothesis:

*Hypothesis 2*: Personality characteristics play a mediating role between cognitive ability and academic performance.

In addition, this study uses psychology health as an intermediary variable to analyze the impact of cognitive ability on academic performance, and how cognitive ability affects academic performance. We propose the following hypothesis:

*Hypothesis 3*: Psychology health mediates between cognitive ability and academic performance.

## Materials and Methods

### Participants

This research was approved by the Research Ethics Committee of the School of Humanities and Social Sciences, University of Science and Technology Beijing, and the data used in the study was provided by the Affiliated Middle School of the University. This study was conducted following the regulations that have been established for human subject protection. This study selected 572 students from a high school in Beijing as the sample, with 291 boys (50.87%) and 281 girls (49.23%). Among the students, there were 225 in the first year (115 boys and 110 girls), 178 in the second year (83 boys and 95 girls) and 169 in the third year (93 boys and 76 girls). As shown in Table 1.

**Table 1:** Distribution of participating students.

Grade	Number of students			
	Boys	Proportion (%)	Girls	Proportion (%)
First grade	115	51.11	110	48.89
Second grade	83	46.63	95	53.37
Third grade	93	55.03	76	44.97
Total	291	50.87	281	49.23

### Procedure

All the tests in this study were conducted on the campus of the Affiliated Middle School of the University of Science and Technology, Beijing. The school teachers uniformly organized all students to enter the computer lab for testing. The test content included questions on middle school level cognitive ability, personality characteristics, and psychology health. The overall duration of the test was 2 h and 30 min.

### Measures

#### Cognitive Ability

The test was conducted using the stimulus information cognitive ability value test system designed by Wo (2010). The test method uses techniques such as subtraction reaction time and addition reaction time (accurate to nanoseconds). Students are provided with visual stimuli, including text, images, and animations. Following this, the total number of fixation points and fixation durations of multiple cognitive areas that emerged from the test students’ feedback are recorded, and the cognitive accuracy of the students is analyzed and tested according to the feedback records. The students are tested for cognitive index value, and cognitive accuracy is obtained through statistical methods to obtain the quantified value of cognitive ability, which is converted into a T-score for the final cognitive ability value. The cognitive abilities tested include five types: MA, IPA, RA, LRA, and TCA. The cognitive ability values obtained by this test method are centered at 100 and have a normal distribution trend in the range of ±50, which has high discrimination validity. Cronbach’s alpha in this study ranged from 0.80 to 0.90.

#### Personality Characteristics

The personality test scale for middle school students was designed by Zhang et al. (2005). The scale has a total of 48 test questions, including four dimensions: planning, self-control, persistence, daring. We set up 12 questions for each one dimensions, and the items were evaluated on a 5-point Likert scale: 1 (very inconsistent), 2 (relatively inconsistent), 3 (uncertain), 4 (relatively consistent), and 5 (very consistent). After accumulation, this is converted into a T-score as the student’s ability value. Though this test, students’ overall academic status can be obtained. The Cronbach *α* coefficient of each dimension of the scale is between 0.60 and 0.93, and the test–retest reliability is 0.85. The validity is 0.91.

#### Psychology Health

The psychology health test scale for middle school students was designed by Pan et al. (2005). The scale has a total of 24 test items, including four dimensions, namely optimism, trust, gregariousness, and enthusiasm. Each dimension is set with six test items. The items were evaluated on a 5-point Likert scale: 1 (very inconsistent), 2 (relatively inconsistent), 3 (uncertain), 4 (relatively consistent), and 5 (very consistent). After accumulation, this is transformed into a T-score as the student’s psychology health value. The Cronbach α coefficient of each dimension of the scale is between 0.79 and 0.91, and the test–retest reliability is 0.81. The validity is 0.87.

#### Academic Performance

This research uses the average of the three most recent test scores of students from the test as their academic performance. Because students choose different subjects, this research only selects the compulsory subjects of Chinese, Mathematics, and English for all students. The results were graded according to the rankings (the lowest score was 0), and the total scores of the three subjects were accumulated.

### Data Analysis

This study first uses Pearson’s correlation analysis to explore the relationship between variables, before using the structural equation model (SEM) to analyze the relationship between cognitive ability, personality characteristics, and psychology health, based on the intermediary analysis process proposed by Wen and Ye (2014). The Bootstrap method is used to test the significance of the mediating role of personality characteristics and psychology health in cognitive ability and academic performance, to obtain the robust standard error and confidence interval of the parameter estimation. If the confidence interval does not include zero, the statistical result is significant (Erceg and Mirosevich, 2008).

## Results

### Common Method Deviation Test

In order to reduce the common method deviations caused by self-reported questionnaires, this study emphasized the authenticity of the answers during the data collection process; the scale and the order of the questions were randomly set for program control. We used Harman’s single factor test to test the effect of program control (Podsakoff et al., 2003), while exploratory factor analysis was conducted on three variables (cognitive ability, personality characteristics, and psychology health) at the same time. It was found that after the rotation, the characteristic roots of eight factors were greater than 1, and the explanatory rate of the first factor was 14.38% (far less than the critical value of 40%), which indicates that the degree of variation in the common method used in this study was within the acceptable range (Wang et al., 2016).

### Descriptive and Bivariate Analyses

The mean values, standard deviations, and intercorrelations of the variables are presented in Table 2. As can be seen from the table, cognitive ability, personality characteristics, psychology health, and academic performance (TS) are all significantly positively correlated, while there are also significant correlations between the sub-items in personality characteristics and psychology health.

**Table 2:** Means, standard deviations, and intercorrelations for variables.

	M	SD	1	2	3	4	5	6	7	8	9	10	11	12	13	14	15	16	17
1. MA	105.112	15.829	1																
2. IPA	102.197	13.246	0.151^**^	1															
3. RA	106.144	7.305	−0.034	0.209^**^	1														
4. LRA	103.138	9.887	0.036	0.076	0.083^*^	1													
5. TCA	94.158	17.286	0.168^**^	0.396^**^	0.340^**^	0.075	1												
6. Planning	101.621	15.934	0.085^*^	0.176^**^	0.117^**^	0.102^*^	0.200^**^	1											
7. Self-control	106.256	15.586	0.310^**^	0.455^**^	0.271^**^	0.222^**^	0.529^**^	0.402^**^	1										
8. Persistence	103.419	15.624	0.219^**^	0.305^**^	0.126^**^	0.145^**^	0.336^**^	0.549^**^	0.592^**^	1									
9. Daring	99.187	16.53	0.131^**^	0.227^**^	0	0.086^*^	0.160^**^	0.339^**^	0.389^**^	0.484^**^	1								
10. Optimism	101.401	15.963	0.066	0.115^**^	0.054	0.079	0.079	0.225^**^	0.281^**^	0.302^**^	0.391^**^	1							
11. Gregariousness	103.025	15.446	0.006	0.163^**^	0.029	0.018	0.105^*^	0.207^**^	0.184^**^	0.221^**^	0.325^**^	0.471^**^	1						
12. Trust	94.55	15.761	0.065	0.177^**^	0.092^*^	−0.002	0.119^**^	0.153^**^	0.251^**^	0.218^**^	0.206^**^	0.441^**^	0.392^**^	1					
13. Enthusiasm	101.233	16.427	0.016	0.141^**^	0.023	0.039	0.126^**^	0.216^**^	0.222^**^	0.232^**^	0.333^**^	0.564^**^	0.745^**^	0.420^**^	1				
14. Chinese	57.818	25.728	0.217^**^	0.509^**^	0.214^**^	0.127^*^	0.586^**^	0.636^**^	0.562^**^	0.662^**^	0.414^**^	0.36^**^	0.42^**^	0.295^**^	0.498^**^	1			
15. Mathematics	50.48	28.912	0.206^**^	0.543^**^	0.238^**^	0.135^*^	0.614^**^	0.661^**^	0.616^**^	0.704^**^	0.451^**^	0.38^**^	0.438^**^	0.315^**^	0.517^**^	0.975^**^	1		
16. English	50.262	28.892	0.199^**^	0.566^**^	0.253^**^	0.136^*^	0.632^**^	0.665^**^	0.645^**^	0.723^**^	0.469^**^	0.391^**^	0.45^**^	0.332^**^	0.532^**^	0.944^**^	0.989^**^	1	
17. TS	159.56	82.698	0.209^**^	0.546^**^	0.238^**^	0.134^*^	0.618^**^	0.661^**^	0.616^**^	0.705^**^	0.450^**^	0.381^**^	0.441^**^	0.318^**^	0.521^**^	0.982^**^	0.999^**^	0.989^**^	1

*N*=572;

* *p*<0.05;

** *p*<0.001.

### Measurement Model Check

Before the mediation effect test, confirmatory factor analysis was needed to test the measurement model. Three latent variables are used in this study, namely cognitive ability (including five indicators of MA, IPA, LRA, RA, and TCA), personality characteristics (including four indicators of planning, self-control, persistence, and daring), and psychology health (including four indicators of optimism, gregariousness, trust, and enthusiasm). The test results show that the model fits well, *χ*^2^(62)=334.72, CFI=0.925, TLI=0.943, SRMR=0.067, RMESA=0.086, and the 90% confidence interval of RMSEA is [0.077, 0.096], indicating that the fitting indicators are all within a good range. Table 3 also shows that the standardized load of each index on the corresponding factor is significant (*p*<0.001).

**Table 3:** Factor loading coefficient table.

Variable	Non-std (Coef.)	SD	*z* (CR)	Std
**Cognitive ability**				
TCA	1.000	–	–	0.632^***^
IPA	0.704	0.072	9.725	0.581^***^
MA	0.419	0.075	5.588	0.288^***^
RA	0.239	0.035	6.743	0.356^***^
LRA	0.175	0.045	3.868	0.194^***^
**Personality characteristics**				
Planning	1.000	–	–	0.574^***^
Self-control	1.345	0.104	12.952	0.788^***^
Daring	0.996	0.096	10.404	0.550^***^
Persistence	1.326	0.103	12.874	0.776^***^
**Psychology health**				
Optimism	1.000	–	–	0.639^***^
Gregariousness	1.215	0.077	15.793	0.810^***^
Enthusiasm	1.436	0.089	16.131	0.901^***^
Trust	0.763	0.071	10.724	0.500^***^

*** *p*<0.001.

### Intermediary Model Checking

**Figure 1:**
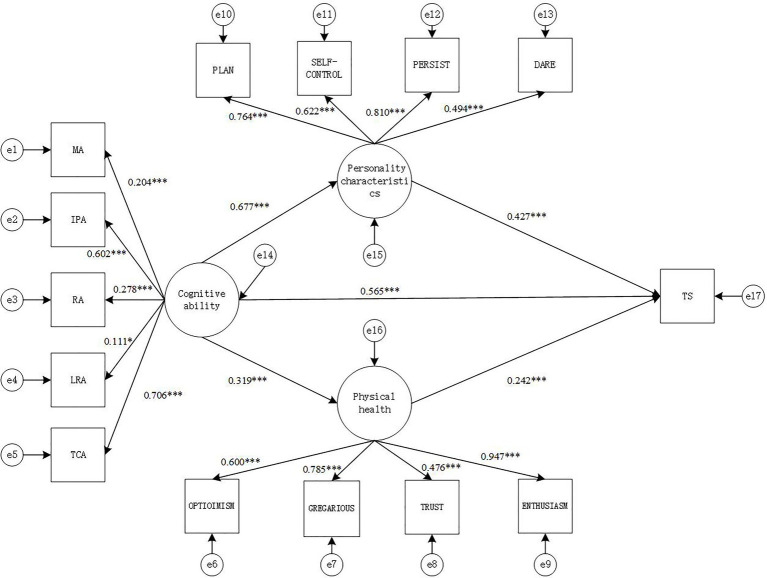
Structural equation intermediary relationship model diagram (model 1). MA, memory ability; IPA, information processing ability; RA, representation ability; LRA, logical reasoning ability; TCA, thinking conversion ability; TS, academic performance. ^*^p<0.05, and ^***^p<0.001.

In this study, the SEM was used to investigate the influence of cognitive ability, personality characteristics, and psychology health on academic performance, and the maximum likelihood estimation method was used to test the hypothesis model in Figure 1.

According to the intermediary analysis process proposed by Wen and Ye (2014), after controlling the influence of gender and age, the SEM is used to analyze the direct effect model of cognitive ability on academic performance. The results show that cognitive ability significantly positively predicts academic performance (*β*=0.873, *p*<0.001). After that, personality characteristics and psychology health were used as intermediary variables along with cognitive ability and academic performance.

#### Model 1: Comprehensive Academic Performance

The fitting index indicators of the SEM were: *χ*^2^(73)=412.35, CFI=0.897, TLI=0.902, SRMR=0.072, RMESA=0.090, the 90% confidence interval of RMSEA is [0.082, 0.099], and the results show that the model fits well.

From the path diagram of the relationship between cognitive ability, personality characteristics, psychology health and Comprehensive academic performance (Figure 1), it can be seen that cognitive ability positively predicts personality characteristics (*γ*=0.677, *p*<0.001) and psychology health (*γ*=0.319, *p*<0.001), while personality characteristics (*γ*=0.976, *p*<0.001) and psychology health (*γ*=0.505, *p*<0.001) both positively predict Comprehensive academic performance. The direct effect of cognitive ability and Comprehensive academic performance is also significant (*γ*=0.565, *p*<0.001).

Based on the mediation model in Figure 1, the non-parametric percentile Bootstrap method is used to further test the significance of the mediating effect of personality characteristics and psychology health. The sampling number is 1,000, and the confidence interval is 95%. The results show that personality characteristics plays Partial mediation effect between cognitive ability and Comprehensive academic performance [mediating effect=0.289, SE=0.054, *p*<0.001, 95% CI=(0.578, 0.775)], while psychology health also plays a role in the relationship between cognitive ability and Comprehensive academic performance, there is Partial mediation between cognitive ability and Comprehensive academic performance [mediation effect=0.077, SE=0.011, *p*<0.001, 95% CI=(0.215, 0.423)]. It can be concluded that personality characteristics and psychology health play a partially mediating role between cognitive ability and Comprehensive academic performance. The mediating effect is (0.289+0.077)/(0.289+0.077+0.565)=0.393 (39.3%).

The Sobel test can also be used to test the significance of the mediation effect. The calculation results are shown in Table 4.

**Table 4:** Sobel test results (model 1).

		*z*	SE	*p*
a_1_	0.677	4.24266762	0.06813614	0
S_a1_	0.061			
b_1_	0.427			
S_b1_	0.093			
a_2_	0.319	4.21188639	0.0183286	0
S_a2_	0.062			
b_2_	0.242			
S_b2_	0.033			

a_1_, regression coefficient of cognitive ability and personality characteristics; s_a1_, corresponding SE; b_1_, regression coefficient of personality characteristics and comprehensive academic performance; s_b1_, corresponding SE; a_2_, regression coefficient of cognitive ability and psychology health; s_a2_, corresponding SE; b_2_, regression coefficient of mental psychology and Comprehensive academic performance; and s_b2_, corresponding SE.

It can be seen from Table 4 that the mediating effect of personality characteristics between cognitive ability and Comprehensive academic performance is significant (*z*=4.24, *p*<0.05). The mediating effect of psychology health between cognitive ability and Comprehensive academic performance is significant (*z*=4.21, *p*<0.05).

#### Model 2: Chinese Academic Performance

The fitting index indicators of the SEM were: *χ*^2^(73)=391.111, CFI=0.893, TLI=0.907, SRMR=0.070, RMESA=0.087, the 90% confidence interval of RMSEA is [0.079, 0.960], and the results show that the model fits well.

**Figure 2:**
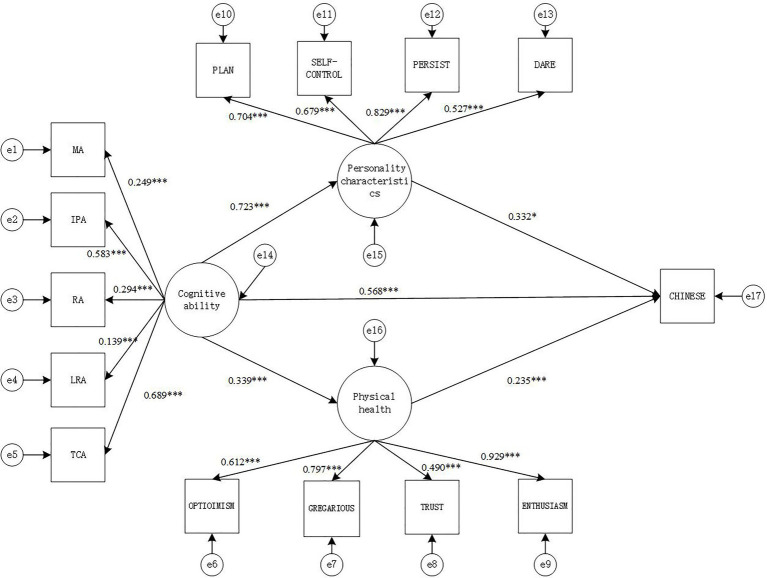
Structural equation intermediary relationship model diagram (model 2). MA, memory ability; IPA, information processing ability; RA, representation ability; LRA, logical reasoning ability; TCA, thinking conversion ability; TS, academic performance. ^*^p<0.05, and ^***^p<0.001.

From the path diagram of the relationship between cognitive ability, personality characteristics, psychology health and Chinese academic performance (Figure 2), it can be seen that cognitive ability positively predicts personality characteristics (*γ*=0.723, *p*<0.001) and psychology health (*γ*=0.339, *p*<0.001), while personality characteristics (*γ*=0.332, *p*<0.05) and psychology health (*γ*=0.235, *p* <0.001) both positively predict Chinese academic performance. The direct effect of cognitive ability and Chinese academic performance is also significant (*γ*=0.568, *p*<0.001).

Based on the mediation model in Figure 2, the non-parametric percentile Bootstrap method is used to further test the significance of the mediating effect of personality characteristics and psychology health. The sampling number is 1,000, and the confidence interval is 95%. The results show that personality characteristics plays Partial mediation effect between cognitive ability and Chinese academic performance [mediating effect=0.240, SE=0.098, *p*<0.05, 95% CI=(0.616, 0.817)], while psychology health also plays a role in the relationship between cognitive ability and Chinese academic performance, there is Partial mediation between cognitive ability and Chinese academic performance (mediation effect=0.079, SE=0.012, *p*<0.001, 95% CI=[0.236, 0.444]). It can be concluded that personality characteristics and psychology health play a partially mediating role between cognitive ability and Chinese academic performance. The mediating effect is (0.240+0.079)/(0.240+0.079+0.568)=0.360 (36.0%).

The Sobel test can also be used to test the significance of the mediation effect. The calculation results are shown in Table 5.

**Table 5:** Sobel test results (model 2).

		*z*	SE	*p*
a_1_	0.723	2.40633065	0.09975188	0.01611367
S_a1_	0.062			
b_1_	0.332			
S_b1_	0.135			
a_2_	0.339	4.09627496	0.01944816	0
S_a2_	0.062			
b_2_	0.235			
S_b2_	0.038			

a_1_, regression coefficient of cognitive ability and personality characteristics; s_a1_, corresponding SE; b_1_, regression coefficient of personality characteristics and Chinese academic performance; s_b1_, corresponding SE; a_2_, regression coefficient of cognitive ability and psychology health, s_a2_, corresponding SE; b_2_, regression coefficient of mental psychology and Chinese academic performance; and s_b2_, corresponding SE.

It can be seen from Table 5 that the mediating effect of personality characteristics between cognitive ability and Chinese academic performance is significant (*z*=2.41, *p*<0.05). The mediating effect of psychology health between cognitive ability and Chinese academic performance is significant (*z*=4.09, *p*<0.05).

#### Model 3: Mathematics Academic Performance

The fitting index indicators of the SEM were: *χ*^2^(73)=408.55, CFI=0.897, TLI=0.902, SRMR=0.071, RMESA=0.090, the 90% confidence interval of RMSEA is [0.081, 0.098], and the results show that the model fits well.

**Figure 3:**
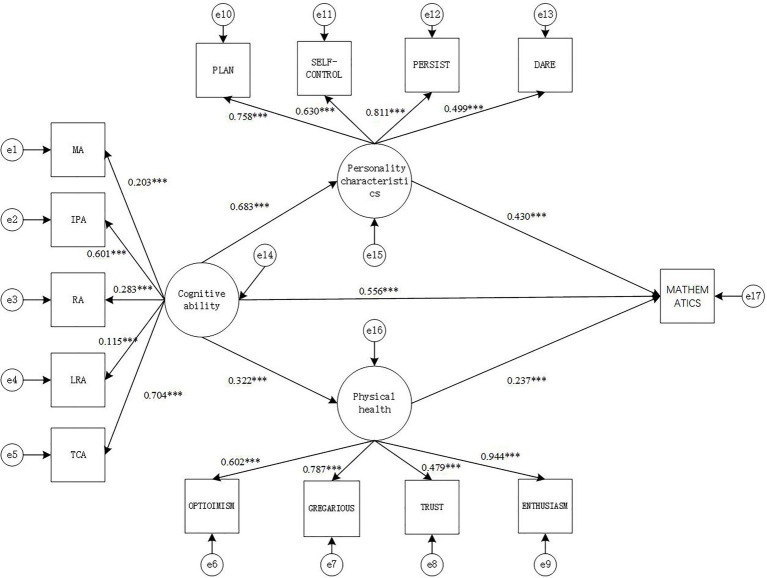
Structural equation intermediary relationship model diagram (model 3). MA, memory ability; IPA, information processing ability; RA, representation ability; LRA, logical reasoning ability; TCA, thinking conversion ability; TS, academic performance. ^***^p<0.001.

From the path diagram of the relationship between cognitive ability, personality characteristics, psychology health and Mathematics academic performance (Figure 3), it can be seen that cognitive ability positively predicts personality characteristics (*γ*=0.683, *p*<0.001) and psychology health (*γ*=0.322, *p*<0.001), while personality characteristics (*γ*=0.430, *p*<0.001) and psychology health (*γ*=0.237, *p*<0.001) both positively predict Mathematics academic performance. The direct effect of cognitive ability and Mathematics academic performance is also significant (*γ*=0.556, *p*<0.001).

Based on the mediation model in Figure 3, the non-parametric percentile Bootstrap method is used to further test the significance of the mediating effect of personality characteristics and psychology health. The sampling number is 1,000, and the confidence interval is 95%. The results show that personality characteristics plays Partial mediation effect between cognitive ability and Mathematics academic performance [mediating effect=0.294, SE=0.057, *p*<0.001, 95% CI=(0.582, 0.780)], while psychology health also plays a role in the relationship between cognitive ability and Mathematics academic performance, there is Partial mediation between cognitive ability and Mathematics academic performance [mediation effect=0.076, SE=0.011, *p*<0.001, 95% CI=(0.218, 0.427)]. It can be concluded that personality characteristics and psychology health play a partially mediating role between cognitive ability and Mathematics academic performance. The mediating effect is (0.294+0.076)/(0.294+0.076+0.556)=0.400 (40.0%).

The Sobel test can also be used to test the significance of the mediation effect. The calculation results are shown in Table 6.

**Table 6:** Sobel test results (model 3).

		*z*	SE	*p*
a_1_	0.683	4.19639532	0.06998626	0
S_a1_	0.061			
b_1_	0.430			
S_b1_	0.095			
a_2_	0.322	4.20844315	0.01813355	0
S_a2_	0.062			
b_2_	0.237			
S_b2_	0.033			

a_1_, regression coefficient of cognitive ability and personality characteristics; s_a1_, corresponding SE; b_1_, regression coefficient of personality characteristics and Mathematics academic performance; s_b1_, corresponding SE; a_2_, regression coefficient of cognitive ability and psychology health; s_a2_, corresponding SE; b_2_, regression coefficient of mental psychology and Mathematics academic performance; and s_b2_, corresponding SE.

It can be seen from Table 6 that the mediating effect of personality characteristics between cognitive ability and Mathematics academic performance is significant (*z*=4.20, *p*<0.05). The mediating effect of psychology health between cognitive ability and Mathematics academic performance is significant (*z*=4.21, *p*<0.05).

#### Model 4: English Academic Performance

The fitting index indicators of the SEM were: *χ*^2^(73)=440.55, CFI=0.897, TLI=0.952, SRMR=0.075, RMESA=0.094, the 90% confidence interval of RMSEA is [0.085, 0.102], and the results show that the model fits well.

**Figure 4:**
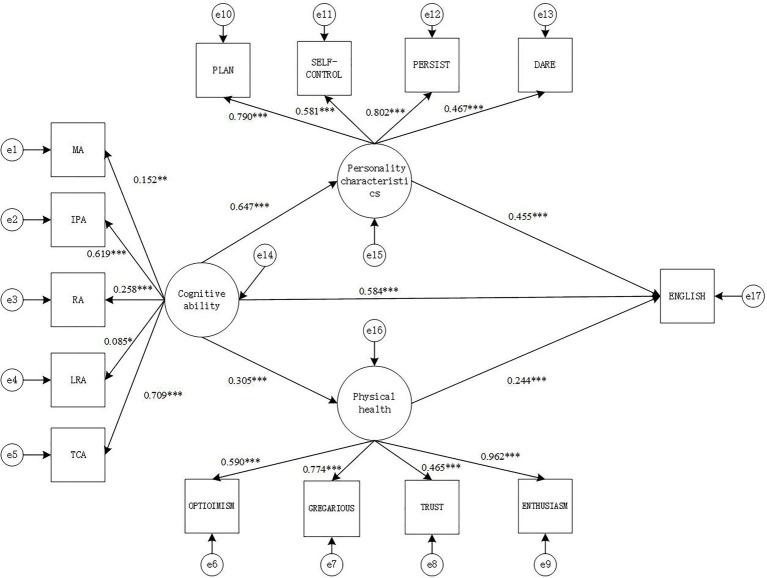
Structural equation intermediary relationship model diagram (model 4). MA, memory ability; IPA, information processing ability; RA, representation ability; LRA, logical reasoning ability; TCA, thinking conversion ability; TS, academic performance. ^*^p<0.05, ^**^p<0.01, and ^***^p<0.001.

From the path diagram of the relationship between cognitive ability, personality characteristics, psychology health and English academic performance (Figure 4), it can be seen that cognitive ability positively predicts personality characteristics (*γ*=0.647, *p*<0.001) and psychology health (*γ*=0.305, *p*<0.001), while personality characteristics (*γ*=0.455, *p*<0.001) and psychology health (*γ*=0.244, *p*<0.001) both positively predict English academic performance. The direct effect of cognitive ability and English academic performance is also significant (*γ*=0.584, *p*<0.001).

Based on the mediation model in Figure 4, the non-parametric percentile Bootstrap method is used to further test the significance of the mediating effect of personality characteristics and psychology health. The sampling number is 1,000, and the confidence interval is 95%. The results show that personality characteristics plays Partial mediation effect between cognitive ability and English academic performance [mediating effect=0.294, SE=0.042, *p*<0.001, 95% CI=(0.545, 0.747)], while psychology health also plays a role in the relationship between cognitive ability and English academic performance, there is Partial mediation between cognitive ability and English academic performance (mediation effect=0.075, SE=0.010, *p*<0.001, 95% CI=[0.205, 0.410]). It can be concluded that personality characteristics and psychology health play a partially mediating role between cognitive ability and English academic performance. The mediating effect is (0.294+0.075)/(0.2940+0.075+0.584)=0.387 (38.7%).

The Sobel test can also be used to test the significance of the mediation effect. The calculation results are shown in Table 7.

**Table 7:** Sobel test results (model 4).

		*z*	SE	*p*
a_1_	0.647	5.06142095	0.05816252	0
S_a1_	0.061			
b_1_	0.455			
S_b1_	0.079			
a_2_	0.305	4.22044278	0.01763322	0
S_a2_	0.061			
b_2_	0.244			
S_b2_	0.031			

a_1_, regression coefficient of cognitive ability and personality characteristics; s_a1_, corresponding SE; b_1_, regression coefficient of personality characteristics and English academic performance; s_b1_. corresponding SE; a_2_, regression coefficient of cognitive ability and psychology health; s_a2_, corresponding SE; b_2_, regression coefficient of mental psychology and English academic performance; and s_b2_, corresponding SE.

It can be seen from Table 7 that the mediating effect of personality characteristics between cognitive ability and English academic performance is significant (*z*=5.06, *p*<0.05). The mediating effect of psychology health between cognitive ability and English academic performance is significant (*z*=4.22, *p*<0.05).

According to the above model, we can find that cognitive ability has a significant effect on academic performance, and personality characteristics and psychology health play a partially mediating role between cognitive ability and English academic performance. The mediation effect is about 40%. Therefore, Hypothesis 2 and Hypothesis 3 are supported (Table 7).

## Discussion

### Impact of Cognitive Ability on Academic Performance

Previous studies have recognized cognitive ability as a psychological feature and a condition for the smooth realization of learning activities (Matthias et al., 2016). High school is a good place for students to effectively improve their learning ability because different subjects have different ability requirements from students. MA can effectively help students improve memory, recitation, and other supporting content. At the same time, it interacts with IPA to improve students’ reading comprehension ability. This is particularly evident in Chinese and English reading. Therefore, in students with good MA, academic performance is also better (Yu et al., 2014).

Representational ability plays an important role in the learning of spatial knowledge in subjects such as mathematics. At the same time, RA can also stimulate associative memory to recite Chinese and English related knowledge, which makes students perform better academically (Lin et al., 2003). TCA is reflected in the speed and accuracy of thought transformation, so any subject learning is inseparable from this ability. Especially in high school mathematics, students with strong TCA can easily summarize and master ideas and methods of completing new math problems, and proficiently apply them to similar problems (Liu, 1988). LRA is divided into two types: inductive reasoning and deductive reasoning. The influence of LRA on academic performance is mostly concentrated in mathematics (Zhu et al., 2020). In recent years, the examination of students in Chinese and English has also been emphasized with the changes in the content of Chinese examinations. Given the logic and rigor of Chinese and English exams (Hu, 2017), LRA has also been shown to affect the scores in the reading part of the Chinese and English exams. IPA is mainly represented by reading comprehension ability and is also related to the efficiency of listening to lectures. Higher IPA fosters in students a greater ability to understand and master reading in the classroom, the formation of knowledge systems, and better academic performance in examinations (Yuan and Wen, 2003).

### The Mediating Role of Personality Characteristics on Cognitive Ability and Academic Performance

Personality characteristics play a complete mediating role in the relationship between cognitive ability and academic performance. Personality internally restricts and determines the unique tendencies and characteristics of individual behaviors (Allport, 1937). Individual learning behavior may be influenced by changes in the environment, but learning activities are inherently guided by stable personality characteristics (Nie et al., 2011). The typical response of personality to situational stimuli is immediate, automatic, emotional, and almost reflexive. But sometimes individuals will use volition control strategies to prevent personality characteristics from triggering stimuli, thereby making an impulsive response. It influences and temporarily changes the individual’s personality characteristics through strategies such as planning, persistence, self-control, and courage to respond to the situation by avoiding some impulsive behaviors, producing other positive behaviors (Mischel, 1983; Mischel and Shoda, 1998). In the analysis of the mediating effect of each sub-item of cognitive ability and personality characteristics, it was also found that self-control in personality characteristics played a complete mediating role between the five cognitive abilities and TS, while planning, persistence, and daring played a full intermediary role. This is because students with high cognitive ability tend to spend less time and get academic satisfaction when completing the same learning task. Therefore, students with high cognitive ability are usually more likely to have higher self-confidence in learning, and are more willing to planning, self-control, and persevere in learning (Liang et al., 2020); similarly, when students with high cognitive abilities come across problems in their studies, they are usually more motivated to solve these problems in order to boost their learning confidence and their sense of accomplishment, and subsequently their own personality characteristics.

Generally speaking, students with higher levels of self-regulation, self-planning, and self-monitoring in their personality characteristics maintain a better and more stable mood when faced with stressful situation. According to the self-determination theory, if students who have the internal and external conditions that satisfy psychological stability (such as self-planning and self-control) can produce behavioral results that promote learning (such as academic performance; Ryan and Deci, 2000). So that students can construct a learning cycle system of planning-execute-persistence-adjustment, so as to carry out learning activities efficiently and achieve better academic performances (Lin, 2020).

### The Mediating Role of Psychology Health Between Cognitive Ability and Academic Performance

Psychology health plays a complete mediating role in the relationship between cognitive ability and academic performance, which also supports the claim that the impact of psychology health on academic performance might be greater compared to cognitive ability (Zeng, 2020). The results of this study are consistent with the formation mechanism of Psychology Health Theory (Zhang et al., 2017). Psychology health is internalized by external stimulation (for example, learning achievement and satisfaction), and the psychological quality closely related to people’s adaptation-development-creation behavior (for example, academic performance), which acts as a “bridge” between cognitive ability and academic performance (Nie et al., 2018). It can be observed that students with high levels of cognitive ability generally have stronger self-management and self-monitoring abilities, and their emotional responses are more moderate than those with average to poor cognitive ability. When students are in an active learning state, it is possible to get a greater sense of achievement from the learning process (Jia et al., 2009). In this way, when students perceive positive academic performance from the outside world, it can not only directly promote students’ academic development, but also enhance students’ psychology health, thus, indirectly improving their academic performances. In addition, easy-going and enthusiastic students generally have good social skills, which helps them maintain a positive learning attitude, and exhibit confidence in the face of academic setbacks and failures (Jia et al., 2009; Fu et al., 2016), which is conducive to improving their academic performance.

### Limitations and Future Directions

One limitation of this study was the small sample size. The next step, for further research, should be to select more schools in other provinces in China for research and comparison. In addition, when considering the factors that affect students’ cognitive abilities, this study only considered the parallel mediating effects between personality characteristics and psychology health, but not the chain mediating effects of personality characteristics and psychology health. We were thus unable to consider the impact of cross-terms on cognitive ability and academic performance. Future studies can focus on this area, for more valuable research results.

## Conclusion

In this study, personality characteristics and psychology health are used as mediating variables between cognitive ability and academic performance. The SEM and the Bootstrap method are used to test the mediating effect. The results of the study showed that cognitive ability has a significant effect on academic performance, and personality characteristics and psychology health play a partially mediating role between cognitive ability and English academic performance. The mediation effect is about 40%.

## Data Availability Statement

The original contributions presented in the study are included in the article/Supplementary Material, further inquiries can be directed to the corresponding author.

## Ethics Statement

The studies involving human participants were reviewed and approved by The Research Ethics Committee of the School of Humanities and Social Sciences, University of Science and Technology Beijing. Written informed consent from the participants’ legal guardian/next of kin was not required to participate in this study in accordance with the national legislation and the institutional requirements.

## Author Contributions

YS and SQ contributed to conception and design of the study. YS contributed to data collection, performed the statistical analysis, and wrote the first draft of the manuscript. All authors contributed to the article and approved the submitted version.

## Funding

This research has received support from the Central University Basic Research Fund of China (FRF-TP-20-084A1).

## Conflict of Interest

The authors declare that the research was conducted in the absence of any commercial or financial relationships that could be construed as a potential conflict of interest.

## Publisher’s Note

All claims expressed in this article are solely those of the authors and do not necessarily represent those of their affiliated organizations, or those of the publisher, the editors and the reviewers. Any product that may be evaluated in this article, or claim that may be made by its manufacturer, is not guaranteed or endorsed by the publisher.
